# Linear Modeling of Neurophysiological Responses to Speech and Other Continuous Stimuli: Methodological Considerations for Applied Research

**DOI:** 10.3389/fnins.2021.705621

**Published:** 2021-11-22

**Authors:** Michael J. Crosse, Nathaniel J. Zuk, Giovanni M. Di Liberto, Aaron R. Nidiffer, Sophie Molholm, Edmund C. Lalor

**Affiliations:** ^1^Department of Mechanical, Manufacturing and Biomedical Engineering, Trinity Centre for Biomedical Engineering, Trinity College Dublin, Dublin, Ireland; ^2^X, The Moonshot Factory, Mountain View, CA, United States; ^3^Department of Pediatrics, Albert Einstein College of Medicine, New York, NY, United States; ^4^Department of Neuroscience, Albert Einstein College of Medicine, New York, NY, United States; ^5^Department of Biomedical Engineering, University of Rochester, Rochester, NY, United States; ^6^Department of Neuroscience, University of Rochester, Rochester, NY, United States; ^7^Centre for Biomedical Engineering, School of Electrical and Electronic Engineering, University College Dublin, Dublin, Ireland; ^8^School of Computer Science and Statistics, Trinity College Dublin, Dublin, Ireland

**Keywords:** temporal response function, TRF, neural encoding, neural decoding, clinical and translational neurophysiology, electrophysiology, EEG, MEG

## Abstract

Cognitive neuroscience, in particular research on speech and language, has seen an increase in the use of linear modeling techniques for studying the processing of natural, environmental stimuli. The availability of such computational tools has prompted similar investigations in many clinical domains, facilitating the study of cognitive and sensory deficits under more naturalistic conditions. However, studying clinical (and often highly heterogeneous) cohorts introduces an added layer of complexity to such modeling procedures, potentially leading to instability of such techniques and, as a result, inconsistent findings. Here, we outline some key methodological considerations for applied research, referring to a hypothetical clinical experiment involving speech processing and worked examples of simulated electrophysiological (EEG) data. In particular, we focus on experimental design, data preprocessing, stimulus feature extraction, model design, model training and evaluation, and interpretation of model weights. Throughout the paper, we demonstrate the implementation of each step in MATLAB using the mTRF-Toolbox and discuss how to address issues that could arise in applied research. In doing so, we hope to provide better intuition on these more technical points and provide a resource for applied and clinical researchers investigating sensory and cognitive processing using ecologically rich stimuli.

## Introduction

**FIGURE 1:**
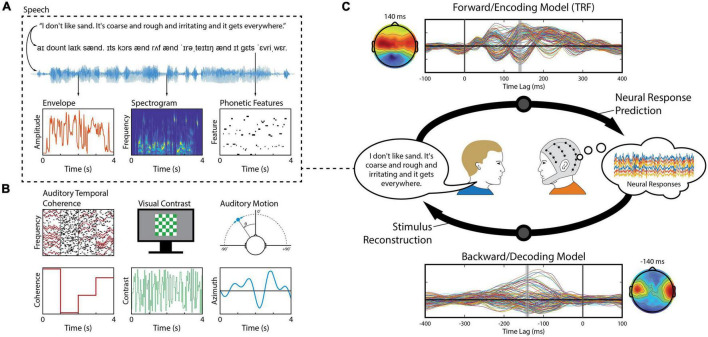
Stimulus features and linear modeling framework. **(A)** A speech signal contains both acoustic and linguistic information and thus can be represented by several different features, such as the envelope, spectrogram, and the timing of phonetic features. Each of these features (or combinations of them) can be used to construct linear models that relate them to the neural activity. **(B)** Aside from speech, linear modeling can be used to quantify responses reflecting, for example, perceptual object formation (O’Sullivan et al., 2015b), visual contrast modulation (Lalor et al., 2006), and auditory motion (Bednar and Lalor, 2018). **(C)** In a series of experiment trials, an observer (right) is presented a stimulus – here, a speaker (left) produces a speech signal (blue time-series shown in panel **A**) – while EEG is recorded simultaneously from their scalp (multi-colored time-series shown in the thought cloud). We can extract any of several features from that stimulus, such as the envelope (red trace in panel **A**). Forward modeling (top arrow) fits a set of weights in an attempt to predict EEG data from a set of stimulus features. Those weights, known as a Temporal Response Function (TRF), are biologically interpretable, akin to a conventional Event Related Potential (ERP). Conversely, backward modeling (bottom arrow) fits a set of weights that map in the reverse direction, known as a decoder, in order to reconstruct a set of stimulus features from the EEG data. While these coefficients are informative, they are not neurophysiologically interpretable in the same way as a TRF.

A core focus of cognitive neuroscience is to identify neural correlates of human behavior, with the intention of understanding cognitive and sensory processing. Such correlates can be used to explicitly model the functional relationship between some “real-world” parameters describing a stimulus or person’s behavior and the related brain activity. In particular, linear modeling techniques have become ubiquitous within cognitive neuroscience because they provide a means of studying the processing of dynamic sensory inputs such as natural scenes and sounds (Wu et al., 2006; Holdgraf et al., 2017; Hamilton and Huth, 2020). Unlike event-related potentials (ERPs) – which are a direct measurement of the average neural response to a discrete event – linear models seek to capture how changes in a particular stimulus dimension or cognitive state are linearly reflected in the recorded brain activity. In other words, we model the outputs as a linear combination (i.e., weighted sum) of the inputs. This enables researchers to conduct experiments using ecologically relevant stimuli that are more engaging and more representative of real-world scenarios. Linear modeling is often used to model neural responses to speech and language (Figure 1A; David et al., 2007; Mesgarani et al., 2008; Lalor and Foxe, 2010; Di Liberto et al., 2015), which will be our focus here, but the same approach can be used to model neural responses to other, non-speech continuous signals (Figure 1B; Theunissen et al., 2001; Machens et al., 2004; Lalor et al., 2009; Gonçalves et al., 2014). The use of such naturalistic stimuli contrasts with current standard practices in which discrete stimuli are presented repeatedly in a highly artificial manner. Moreover, the simplicity of linear models enables researchers to interpret the model weights neurophysiologically, providing insight into the neural encoding process of naturalistic stimuli (Haufe et al., 2014; Kriegeskorte and Douglas, 2019).

The uptake in linear modeling techniques in cognitive neuroscience has led to a similar adoption in the applied and translational neurosciences. This has greatly facilitated the study of naturalistic sensory processing in various clinical cohorts such as individuals with hearing impairments (Somers et al., 2018; Decruy et al., 2020) autism spectrum disorder (Frey et al., 2013), schizophrenia (Lalor et al., 2008) and dyslexia (Power et al., 2013; Di Liberto et al., 2018b), as well as studying sensory processing deficits in older adults (Decruy et al., 2019; Anderson and Karawani, 2020; Broderick et al., 2021). However, studying clinical cohorts raises important issues when constructing and interpreting linear models. For example, particular care is required when performing group comparisons of model weights and evaluating model performance, as will become evident as we elaborate on the numerous factors influencing data and model integrity below. Furthermore, linear modeling poses challenges and considerations that are not typical for other types of electrophysiology analysis. As a model, it is meant first and foremost to quantify the functional relationship between the stimulus features of interest and the recorded neural response. Modeling electrophysiological data is non-trivial because neighboring time samples and channels are not independent of each other, thus standard methods for quantifying the significance of the fit cannot be used. Furthermore, the interpretation of the results must take into careful consideration the particular preprocessing steps applied, which can have major effects on the response patterns obtained using linear models, especially with respect to filtering, normalization and stimulus representation (Holdgraf et al., 2017; de Cheveigné and Nelken, 2019). Here, we wish to provide guidance and intuition on such procedures and, in particular, to promote best practices in applying these methods in clinical studies.

In this review, we will step through the stages involved in designing and implementing ecological neuroscientific experiments with linear modeling in mind. First, we discuss experimental design considerations for optimizing model performance and ecological relevance. Second, we discuss data preprocessing and stimulus feature extraction techniques relevant to linear modeling. Third, we discuss the various model design choices and their use cases. Fourth, we review how to appropriately train and test linear models, as well as evaluate the significance of model performance. Fifth, we discuss considerations for comparing models generated using different stimulus representations. Sixth, we discuss the considerations when interpreting linear model weights. Finally, we discuss what can go wrong when using linear models in applied cognitive neuroscience.

In each section, we will also introduce issues that are relevant to clinical research via a hypothetical clinical experiment. Because linear modeling is commonly used to study the neural processing of natural speech (for reviews, see Ding and Simon, 2014; Holdgraf et al., 2017; Obleser and Kayser, 2019), the example experiment will also focus on speech processing, but the methods we describe generalize to many other stimulus types, paradigms and participant groups. The researcher should modify the experimental design, data preprocessing and model design steps according to their own research questions. Likewise, our focus will be on the linear modeling of EEG data, but these methods can be applied to other types of neurophysiological data, such as MEG, ECoG, fMRI, and fNIRS. When discussing model design and implementation, we will make specific reference to the mTRF-Toolbox, an open-source MATLAB package available on GitHub^1^. All functions referenced in this article were from version 3.0. While we do not elaborate on the technical details of the mTRF-Toolbox (for that we point the reader to Crosse et al., 2016a), we do provide example code in the highlighted boxes and briefly walk the reader through its implementation.

### Example Experiment

Suppose we observe that individuals in a particular clinical group display a specific behavioral deficit in the processing of speech sounds (i.e., a phonological deficit), whilst appearing to have intact general acoustic processing. We hypothesize that the observed phonological deficit can be explained by weaker phonetic encoding.

To test our hypothesis, we plan to measure how well phonetic features are represented in the ongoing neural activity of our clinical participants compared to a control group. Specifically, we will quantify the predictive power of models that uniquely represent phonetic and acoustic processing in each group. We hypothesize that the predictive power of the phonetic model will be relatively reduced in our clinical cohort, reflective of impaired neural encoding of phonetic features and potentially underpinning their behavioral deficit, whereas the performance of the acoustic model will be comparable between groups. To be clear, the example experiment we discuss in this paper is made up for didactic purposes and the data were artificially generated for illustrative purposes.

## Experimental Design

One of the benefits of employing linear models for EEG analysis is the ability to use dynamic and naturalistic stimuli (Hamilton and Huth, 2020). Additionally, it allows the researcher to study sensory processing in an ecologically relevant context, providing them the opportunity to design experiments that are more engaging for the participants. This can potentially improve the quality of the data collected, as well as the reliability of the researcher’s findings. The following considerations should be taken into account when designing ecological neuroscientific experiments.

### Use Subject-Relevant Stimulus Material

This is primarily relevant to speech studies and is important for ensuring subject compliance with the task, particularly when studying younger cohorts and individuals with neurological disorders or developmental disabilities. For example, it is important when choosing an audiobook or movie that it is:

(1) Age-relevant (e.g., a children’s story versus an adult’s podcast),
(2) Content-relevant (a quantum physics lecture may not be everyone’s cup of tea), and
(3) Language-relevant (speaker dialect and even accent may impact early stage processing across participants/groups differentially).

It may in some situations be necessary to create such content from scratch by recording a native speaker reading the chosen material aloud. However, there are also publicly available stimulus databases such as MUSAN: an annotated corpus of continuous speech, music and noise (Snyder et al., 2015), and TCD-TIMIT: a phonetically rich corpus of continuous audiovisual speech (Harte and Gillen, 2015).

### Use a Well-Balanced Stimulus Set

It is important to consider the frequency of occurrence of particular stimulus features that are relevant to the study (e.g., spectral or phonetic features). For example, choosing stimulus material that contains only a few instances of particular phonemes will make it difficult to reliably model the neural response to such phonemes without overfitting to the noise on those examples. This can be avoided by employing phonetically balanced stimuli, such as the aforementioned TCD-TIMIT corpus (Harte and Gillen, 2015), or in a *post hoc* manner by focusing the analysis on a subset of the data, i.e., only the features that are equally represented or only the time segments where the stimuli are well balanced. It is also best to work with longer stimuli that are preferably broadband or quasi-periodic (e.g., speech or music recordings). Linear modeling can produce ambiguous results if the stimulus is perfectly periodic since periodicity can result in artificially periodic-looking evoked responses which can also increase difficulties with quantifying the accuracy of the model.

In addition, to build models that generalize better to a wider variety of stimuli, one might consider how to incorporate more low-level features in their stimuli. For speech studies, additional acoustic variability may be desirable, independent of the linguistic content. This could be accomplished by including multiple speakers with substantially different spectral profiles (e.g., both male and female speakers), as well as speakers who provide a more dynamic range in prosody and intonation across the speech content (e.g., trained actors or media presenters). Models that are trained on a broader range of stimuli are less likely to overfit to stimulus features that are not of interest to the researcher (such as speaker identity, sex, or location), but may perform slightly worse on average. Such decisions should be based on the researcher’s overall goals.

Another important consideration is the presentation order of the stimuli. For example, the experimenter may wish to present the various chapters of an audiobook in their natural order, thus allowing the participants to follow the storyline and making the experiment more engaging. However, it is important to remember that such experiments lead to neural responses that are specific to the fixed presentation order, which could be an issue when considering certain types of classification analyses (Li et al., 2020). While the possibility of presenting audio stimuli (e.g., audiobooks, music) with long-term dependencies is an advantage of this modeling approach rather than an issue, we suggest designing experiments with several short audio stimuli (e.g., 5-min stories), thus allowing for a randomization of the stimulus order and for an alternation of other parameters, such as the speaker or musical instrument.

When considering your stimuli, we also suggest adopting an open mind with respect to possible future studies. Choosing materials that are rich in other features that can be modeled (e.g., semantic content, prosody, temporal statistics) can provide fruitful opportunities for re-using your data to tackle new questions beyond those planned in your current study (fans of Dr. Seuss and James Joyce beware!).

### Collect Enough Training Data

To avoid building models that overfit to specific features or noise in your data, it is crucial to consider how much training data is required, or in other words, how much novel stimulus material it is necessary to have for the experiment. For most purposes, we recommend collecting a minimum of 10 to 20 min of data per condition, although more data may be required for larger, multivariate models (e.g., spectrogram) or when features are sparsely represented (e.g., word onsets). While it is feasible to construct high-quality models from many short (<5 s) stimulus sequences, such as individual words or sentences, it is preferable to use longer (>30 s) stimulus passages because it reduces the number of onset responses in the neural data, which tend to be larger and obscure feature-specific responses of interest (for tips on avoiding this, see *Data Preprocessing*).

While more data is always desirable for training good models, be aware that longer recording sessions can cause subject fatigue, particularly in children, older adults, or clinical cohorts. Reduced concentration or attention can negatively impact the neural tracking of stimuli, leading to poor model performance (Ding and Simon, 2012; O’Sullivan et al., 2013, 2015a). Recording time can be shortened without compromising the quality of the model by:

(a) Removing silent periods in the speech stimuli greater than a certain duration (e.g., >300 ms; Ding and Simon, 2013) in order to maximize the stimulus information rate (but see *Further Considerations*), or
(b) Using subject-independent models, i.e., models constructed from the pooled data of multiple subjects (see *Model Design*; Di Liberto and Lalor, 2017).

It is also important to consider the issue of subject fatigue when deciding how many experimental conditions to include in your paradigm. When a large number of conditions is unavoidable (e.g., multisensory or speech-in-noise studies), we recommend splitting the data recordings into multiple sessions, as within-subject inter-session reliability is typically very good for such models^2^. Overall, the researcher should judiciously balance the tradeoff between data quantity and quality to suit their specific needs.

Another consideration with regard to data quantity is how many neural recording channels to use. In the case of ECoG or MEG this is often predetermined by the surgery or equipment respectively. Most fMRI studies involve whole brain scans, so, again, the number of channels (i.e., voxels) is predetermined. That said, for studies that require higher temporal or finer local spatial resolution, scanning protocols can be adapted to focus on a more limited number of voxels. In the case of EEG, if there are no mitigating factors, one may wish to collect data from as many electrodes as one has available. Indeed, collecting more channels is certainly advantageous when it comes to exploiting redundancy across channels as part of preprocessing, and for carrying out certain types of analysis such as source estimation. However, when using forward models (see *Model Design*) to test specific hypotheses – and especially where time might be a concern given the particular cohort under study (e.g., infants) – it may be favorable to use fewer channels. For example, if one was interested in studying the neural correlates of semantic dissimilarity in language, one might expect the relevant responses to be located over midline parietal scalp (Broderick et al., 2018), in line with the long history of work on the N400 component (Kutas and Federmeier, 2011). And, given that forward models are derived channel-by-channel, one could choose to record from only a limited set of channels that give coverage of midline parietal scalp. For backward models (see *Model Design*), in general, the more channels the better (but see *Further Considerations*). This is because the analysis is multivariate, thus the resulting model can simultaneously utilize relevant information across multiple channels. As such, the model learns the optimal weightings to apply to each channel and can ignore (i.e., down-weight) irrelevant channels. That said, where issues of time, portability, and/or limited channel availability are relevant, one may be advised to choose fewer channel locations to achieve a balance between those channels that are likely to strongly represent the signals of interest and other, more distant channels that can provide both a reference and a clean signature of background EEG that may be common across channels.

### Use Active Task Designs

Although naturalistic stimuli tend to be inherently more engaging than artificial stimuli, the use of prolonged natural stimulus sequences can still induce fatigue, which can negatively impact data quality and results. To ensure continuous engagement with the stimulus content, we recommend including an appropriate behavioral task. This could consist of:

(a) Answering comprehension questionnaires immediately after the end of each trial,
(b) Recalling the last sentence at random intervals during the trial, or
(c) Detecting intermittent targets or anomalies in the stimulus, e.g., respond to a certain word, nonsense word or acoustic perturbation (see *Further Considerations*).

The other advantage of including a task is that, in addition to potentially improving the quality of the data, the researcher will have valuable behavioral data to go with it, such as measures of comprehension, intelligibility, detection accuracy or response time. Even tasks that are inherently active, such as auditory attention experiments, can be greatly enhanced by the addition of appropriate psychophysical tasks.

### Example Experiment

With these considerations in mind, we chose for our example experiment a professionally recorded audiobook featuring a Sherlock Holmes adventure, read by a female actor. The story lasted approximately 30 min, providing a sufficient amount of EEG data per subject with which to fit our models. The stimuli are presented to our subjects in 2-min-long continuous passages (15 × 2-min trials in total), striking a nice balance between reducing onset effects whilst still providing regular breaks in order to minimize subject fatigue and discomfort. During stimulus presentation, we record 128-channel EEG data (plus two mastoid channels) at a sample rate of 512 Hz. Based on a power analysis, we collect these data from 30 individuals in our clinical cohort and 30 sex, age, and IQ-matched control subjects.

### Further Considerations

While reducing silent intervals above a certain duration in speech stimuli can increase the information rate and reduce the overall experiment time, previous work has shown that manipulating the statistical distribution of pauses in speech, such that the local speech rate is irregular, can reduce neural entrainment to rhythmic auditory features in the delta (<4 Hz) frequency band (Kayser et al., 2015). Thus, while increasing the rate of speech (to a certain degree) does not appear to impact behavior, the neural correlates of speech processing in specific frequency bands (i.e., delta) are impacted and may not accurately reflect the typical underlying neurophysiology of speech processing in a naturalistic or real-world context. It is also important to consider how different subject cohorts may be differentially impacted by such manipulations, potentially leading to false conclusions.

While the general rule is that it is better to have more neural recording channels for decoding stimulus features from neural data (i.e., for backward modeling), a recent study demonstrated in 90 subjects that the optimal number of EEG electrodes is approximately 20 well-positioned electrodes (Montoya-Martínez et al., 2021). Specifically, the analysis started with a higher density (64-channel) setup, and each recording channel was systematically removed one-by-one to evaluate their influence on reconstruction error via a utility metric that was based on the correlation between the true and reconstructed signal (Bertrand, 2018). Thus, if the researcher has the ability to record with a high-density, then it is advisable to do so as the montage can always be reduced offline to utilize the best set of channels.

It is important to employ an appropriate task in the context of your experiment to avoid the subject engaging with the wrong aspect of the stimulus. For example, if we wish them to engage with the language content of a speech stimulus, then using a speech-specific target, such as a content or nonsense word, is better than them listening for an acoustic anomaly such as a perturbation in frequency or amplitude.

## Data Preprocessing

Prior to constructing our model, it may be necessary to clean and preprocess the neural data. In our experience, linear modeling is fairly robust to sparse artifacts, such as eyeblinks, which are usually uncorrelated with the stimulus features of interest. However, particularly noisy data can be difficult to work with and neural recordings such as EEG are notoriously noisy. On the other hand, aggressive filtering of the data is unfortunately common and can exacerbate existing artifacts, producing spurious oscillatory modulations or ringing in the time domain (for an in-depth review, see de Cheveigné and Nelken, 2019). In general, we recommend a more conservative preprocessing strategy to avoid this, but also make suggestions for a more liberal approach as needed for noisier datasets and less cooperative cohorts (e.g., infants or certain clinical groups). Ideally, a single set of preprocessing parameters – defined by the noisiest cohort/dataset – should be applied to all participants within a given study.

In this section, we focus on the preprocessing steps relevant to modeling multivariate stimulus-response data. Of course, there are other rudimentary preprocessing steps necessary for certain types of data, such as EEG, MEG, ECoG, and fMRI, for which the reader must consult the necessary literature (e.g., Hämäläinen et al., 1993; Bigdely-Shamlo et al., 2015; Stolk et al., 2018; Esteban et al., 2019). The following preprocessing steps apply in general and should be implemented in order:

1. High-pass filter (HPF) the data to remove any unwanted DC shifts or slow drift potentials (<1 Hz) that may be present, for example, due to amplifier DC components or electrode junction potentials caused by sweating (applies to EEG). Note, higher-order filters with sharp roll-offs can introduce significant artifacts in the time domain and thus should be avoided (de Cheveigné and Nelken, 2019). Additionally, we recommend using zero-phase-shift filters to avoid introducing temporal lags between the stimulus and response. However, zero-phase filters are non-causal, which can affect the interpretation of model weights at lags prior to zero (see *Interpretation of Model Weights*, and de Cheveigné and Nelken, 2019). This is particularly problematic for modeling auditory brainstem responses (ABRs) which occur at short latencies, thus it is recommended to use a low-order causal filter (see Maddox and Lee, 2018). For cooperative participants, we recommend using a HPF with a cutoff frequency in the range [0.1, 0.5] Hz (order ≤ 3). For less cooperative participants, we recommend a cutoff frequency in the range [0.5, 1.0] Hz (order ≤ 5). Note, for modeling the stimulus-response relationship at specific neural frequency bands (e.g., theta, low gamma), one will often use much higher HPF cutoffs as part of a band-pass filter.
2. Low-pass filter (LPF) the data to remove any unwanted high frequency noise that may be present, for example, due to muscle contractions or environmental interference such as 50/60-Hz line noise. Alternatively, if the researcher wants to retain high-frequency information, line removal can be implemented using a notch filter or a method such as ZapLine (de Cheveigné, 2020). Linear modeling tends to automatically filter the data in such a way that focuses on the most relevant energy range (usually lower-frequencies) for stimulus-based prediction (de Cheveigné et al., 2018); however, an LPF step can help improve model performance, particularly for noisier datasets. Similar to HPF, we also recommend using zero-phase filters. For cooperative participants, we recommend using a LPF with a cutoff frequency in the range [20, 40] Hz (order ≤ 3). For less cooperative participants, we recommend a cutoff frequency in the range [10, 30] Hz (order ≤ 5). Again, for modeling the stimulus-response relationship at specific neural frequency bands (e.g., delta, theta), one may wish to use much lower LPF cutoffs as part of a band-pass filter. Note, for modeling ABRs, we recommend using a much higher LPF cutoff (e.g., ∼2000 Hz) and removing the line noise using a notch filter instead (see Maddox and Lee, 2018).
3. Remove or interpolate any bad recording channels identified as having (a) relatively high or low variance, (b) excessive noise/artifacts, or (c) that have been compromised due to bridging (applies to EEG). We recommend limiting channel interpolation to <10% of all recording channels, any more than that may warrant discarding the entire segment of data altogether. As a sanity check, if the newly interpolated data are highly correlated with the original data (e.g., Pearson’s *r* > 0.7), then the original data may in fact be valid and worth retaining.
4. Re-reference the data to an appropriate channel(s) to enhance neural activity in a region of interest or recover the common mode rejection ratio (necessary for EEG systems such as BioSemi). While model prediction scores may be unaffected by re-referencing, it can enhance the interpretation of the model weights when done correctly. For EEG, mastoid references tend to emphasize responses to auditory stimuli over fronto-central scalp while more frontal references tend to emphasize responses to visual stimuli over occipital scalp. Alternatively, an average reference (i.e., the mean of all recording channels) is a good choice if one does not want to emphasize the activity of a particular region over another, as might be the case in multisensory experiments (for review, see Murray et al., 2008). Note, when using an average reference, ensure that any bad channels are already removed or interpolated (see step 3).
5. Downsample the data to reduce computation time during model training, particularly for larger, multivariate datasets. Prior to downsampling, low-pass filter the data well below the Nyquist frequency (i.e., below half the desired sample rate) to prevent temporal aliasing. Note that some downsampling functions have built-in anti-aliasing filters (e.g., resample() in MATLAB). If an LPF is applied in step 2, it can function as an anti-aliasing filter, provided its cutoff is below the Nyquist frequency; an additional antialiasing filter constitutes an unnecessary filtering operation that introduces additional filtering artifacts. After downsampling, the sample rate should be at least 2 times the highest frequency of interest. For quantitative analyses at low frequencies, such as predicting delta- or theta- frequency responses, it is possible to downsample the data to sample rates as low as 40 Hz (e.g., Ding and Simon, 2013). Furthermore, most of the signal detected by standard EEG recording systems is below about 25 Hz^3^. For more qualitative analyses, such as interpretating the model weights, a higher resolution of >100 Hz may be desired for visualization purposes.
6. [OPTIONAL] For non-invasive recordings such as EEG/MEG, sparsity-driven artifact rejection methods, such as independent components analysis (ICA), can be used to remove distinct artifacts such as eye-blinks, facial and neck movements, and line noise (see Hyvärinen and Oja, 2000). While this step is not always necessary because such artifacts are typically sparse and uncorrelated with the stimulus features of interest, it may be necessary for noisier datasets or less cooperative subjects. Alternatively, linear denoising source separation methods based on the reproducibility of the response to a repeated stimulus, such as joint decorrelation (de Cheveigné and Parra, 2014), can be used to enhance the underlying stimulus-driven EEG signal. However, one may wish to avoid using repeated stimuli in an effort to maximize ecological validity and minimize the effects of prior knowledge. If so, linear denoising approaches that extract neural components common to a group of subjects can be used instead, such as multiway canonical correlation analysis (de Cheveigné et al., 2019).
7. Remove the first 500 to 1000 ms of data at the start of every trial to avoid fitting the model to the neural response elicited by the onset of stimulation. Such onset responses tend to be greater in magnitude than those that track ongoing modulations in certain stimulus parameters, obscuring the neural activity of interest. For shorter (<5 s) stimuli, this may not be feasible but can be circumvented by using stimuli with less abrupt onsets that ramp up gradually in intensity, or by including additional stimulus features that account for the variance explained by the onset, such as impulse trains that represent the acoustic onsets in speech (Brodbeck et al., 2020).
8. [OPTIONAL] Normalizing the neural data is not always necessary but there are certain situations where it is useful. For example, normalizing the data leads to more consistent tuning of model parameters across datasets (see *Regularization* in *Model Training and Testing*). This is particularly important when working with subject-independent models because they are trained on pooled data from multiple subjects (see *Model Design*). A common normalization technique is to standardize (or z-score) the data by subtracting its mean and dividing by its standard deviation. To ensure consistency across trials, all data within the same subject should be normalized together, not separately (i.e., using a global measure of mean and standard deviation). However, one may wish to omit the test data to avoid biasing the training set. Similarly, multichannel neural data should be normalized together in order to preserve the relative power across channels, instead of normalizing channel-by-channel. Normalization of the data can also be used to obtain meaningful units of measure when computing model weights, for example, by scaling EEG data by the amplifier microvolts/bits conversion ratio to obtain microvolt units (see Lalor et al., 2006). This step requires careful quantification of certain physical stimulus parameters and must be implemented jointly with the corresponding stimulus normalization step described in *Stimulus representation*. Thus, such normalization procedures should only be attempted if deemed completely necessary.

### Example Experiment

The preprocessing strategy chosen for our example experiment takes into account the relevant speech electrophysiology literature; natural speech is encoded in the EEG signal primarily in the delta (0.5–4 Hz) and theta (4–8 Hz) frequency bands (Poeppel, 2003; Giraud and Poeppel, 2012; Ding and Simon, 2014). We initially filter our EEG data between 0.5 and 40 Hz using a 3rd order Butterworth filter. Note, such conservative filtering is considered a preprocessing step and leaves open the possibility of filtering the data further to explore specific hypotheses about how neural activity in different frequency bands (e.g., delta, theta, low gamma) might differentially reflect the processing of certain features of speech (Ding et al., 2014; Di Liberto et al., 2015). Next, we detect any bad channels based on two criteria: channel variance and inter-channel correlation. Channels with values more than 3 standard deviations from the mean are recalculated by spline-interpolating the nearest 6 channels on every trial. After channel rejection, we re-reference the EEG to the average of the two mastoid channels. Finally, given that we have filtered our data below 40 Hz, we downsample our data from 512 to 128 Hz in order to reduce model training time.

### Further Considerations

These preprocessing steps should be conducted on the entire dataset prior to segmenting it into individual trials (i.e., before extracting the minute-long epochs time-locked to the stimulus triggers). Filtering after data segmentation can introduce edge artifacts that could masquerade as large onset/offset responses and impact subsequent analyses. However, if the neural data consists of discontinuous trials (i.e., in separate files or pauses in the recording between trials), then filtering should be done separately on each trial because discontinuities at trial boundaries can also introduce filter artifacts. Note, artifact rejection, such as ICA, should be done collectively by concatenating all files belonging to the same recording session.

When using a conservative HPF cutoff (e.g., ≤0.1 Hz), you may wish to first conduct ICA on data that was filtered using a higher cutoff (e.g., >1 Hz), and then apply the corresponding ICA weights to the 0.1-Hz HPF data. The reason for this being that ICA works best on data filtered above 1 Hz because it can become biased toward lower frequencies which tend to have greater power and can be contaminated by electrode junction potentials (see Winkler et al., 2015).

## Stimulus Representation

Linear modeling requires the quantification of specific stimulus features that relate to the corresponding neural data in some meaningful way. Designing stimulus features is an important step in the overall process and comes with its own underlying assumptions. Ultimately, these choices define our hypothesis about how the stimulus is represented in the neural activity, and it will determine the stage of processing along the sensory or cognitive pathway represented by the resulting model.

### Choosing Stimulus Features

**FIGURE 2:**
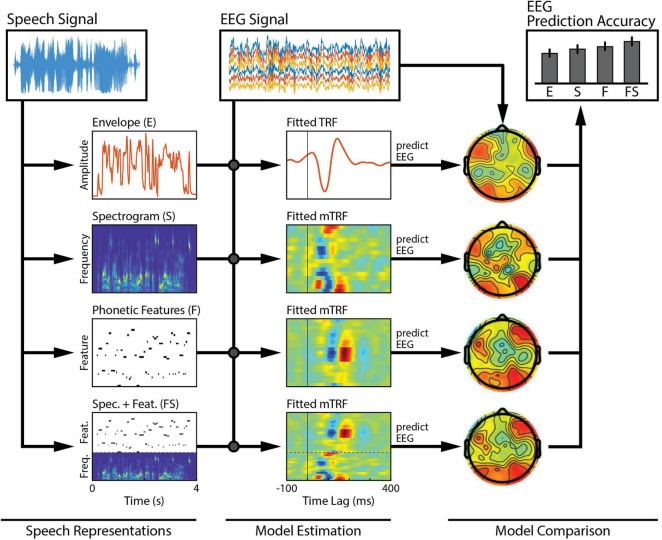
Comparison across a family of models. Several features of interest are derived from our speech signal. Each of these features (or combinations of them) can be regressed against brain activity to estimate single- or combined-feature encoding models (TRFs). The TRFs can be used to predict held-out EEG data, channel-by-channel, and the accuracy of those predictions can be measured to assess the quality of the model. Here, we compare model performance across a set of six bilateral auditory-responsive electrodes (Di Liberto et al., 2015).

Stimulus features are derived from some physical or perceptual parameters that describe the stimulus. Examples of physical parameters range in complexity, from intensity of acoustic vibrations and pixel luminance, to motion and spectral content (Figures 1A,B). Perceptual parameters are more abstract and often relate to how such physical parameters are mapped perceptually to categorical attributes, such as words or objects. A linear model is defined by its choice of stimulus features as they determine the level of processing along the sensory or cognitive pathway to be modeled. Such low-level and high-level stimulus features can also be combined to create richer stimulus representations that span multiple processing stages and explain more of the variance in the neural activity (e.g., Di Liberto et al., 2015; O’Sullivan et al., 2017; Figure 2). See also *Further Considerations*.

### Non-linear Transformations

Another important factor to consider when defining stimulus features is that it provides an opportunity to apply biologically relevant non-linear transformations that would otherwise be overlooked by the computations of a linear model. For example, many aspects of human auditory perception, such as loudness and pitch, bear a logarithmic relationship to the corresponding physical properties of sound (Stevens, 1955). Such non-linear relationships between the raw audio signal and the related brain activity can be explicitly incorporated into a linear model by applying an appropriate logarithmic scaling to the relevant stimulus features (see Box 1). This yields a more biologically plausible model that still benefits from the efficiency and interpretability of linear computations. Moreover, applying such power laws has been shown to improve the ability of linear models to decode auditory attention (Biesmans et al., 2016). We will step through these considerations in our example experiment, but such parameterizations will be up to the researcher who should consider their own expectations based on the relevant literature, and an appropriate, and not overparameterized, representation of the features of interest. Considerations here will affect how we interpret the weights of our model (for further discussion, see *Interpretation of Model Weights*).

**Box 1. Speech envelope implementation.**

A common feature that is often used to model brain responses to natural speech is what’s known as the speech envelope (sometimes referred to as the temporal or amplitude envelope). The speech envelope represents the intensity of the speech signal as a function of time, and comprises energy changes corresponding to phonemic and syllabic transitions. Fortunately, envelope frequencies that are important for speech intelligibility (2–20 Hz) happen to reside within the frequency range of typical EEG recordings (Drullman, 1995).

In mTRF-Toolbox, the speech envelope can be extracted directly from an original audio signal, sampled at *f*_*sin*_, by computing the root-mean-square (RMS) over a window of samples and logarithmically scaling the resulting RMS intensity (Lalor and Foxe, 2010). The resulting envelope can be computed at the original sample rate or downsampled to a desired rate, *f*_*sout*_.

envelope = mTRFenvelope(audio,fsin,fsout,window,comp);

The function automatically calculates the number of samples over which to compute the RMS intensity based on the values of fsin and fsout. Setting the window parameter to values greater than 1 will apply additional smoothing to the envelope. To model human hearing, compression can be applied to the envelope using the comp parameter, which raises the RMS intensity to the power specified. It is common to use a power value of 0.3 (Biesmans et al., 2016). In addition to this, more sophisticated transformations can be applied to model the peripheral and early auditory system using MATLAB packages such as Auditory Toolbox (Slaney, 1998), NSL Auditory-Cortical MATLAB Toolbox (Ru, 2001) and The Auditory Modeling Toolbox (Majdak et al., Submitted).

### Normalization

Normalization of stimulus features is a common preprocessing step when modeling stimulus-response data. It ensures consistent scaling of the resulting model weights, as well as consistent tuning of model parameters across datasets (see *Regularization* in *Model Training and Testing*). Similar to neural data, stimulus features can be z-scored or even just normalized by their standard deviation to maintain positive values. To ensure consistency across trials, all stimuli should be normalized together, not separately (i.e., using a global measure of mean and standard deviation). Similarly, multivariate stimulus features should be normalized together in order to preserve the relative power across features. However, if such differences are not meaningful (i.e., not representative of physical or biological parameters), normalization can be done on each feature separately to reduce their influence on the magnitude of feature weights (Holdgraf et al., 2017). Normalization of stimulus features can also be used to obtain meaningful units of measure when computing model weights, for example, by scaling the intensity feature by the frame rate at which the stimulus was presented to obtain microvolt units (see Lalor et al., 2006). This normalization technique requires careful quantification of certain physical stimulus parameters and must be implemented jointly with the corresponding EEG normalization step described in *Data Preprocessing*. Thus, such normalization procedures should only be attempted if deemed completely necessary.

#### Example Experiment

In this experiment, we want to compare acoustic and phonetic processing between our groups. The first step is to isolate the particular feature of interest from the stimulus: the “acoustics,” which will be represented by the speech envelope and spectrogram, and the “phonetics,” which will be represented by the timing of phonetic features. In general, it is advisable to consult the literature on this and describe the method of extracting the features in as much detail as possible when reporting the results. In our example, we will use feature extraction methods based on the speech EEG literature. To approximate the spectrogram, the stimulus will be filtered into 32 logarithmically spaced frequency bands using a gammachirp filterbank to model human auditory frequency perception (Irino and Patterson, 2006). The narrowband envelopes are then computed by taking the moving root-mean square (RMS) over windows of 250 samples to downsample the audio from 16 kHz to 128 Hz to match the rate of the EEG (Lalor and Foxe, 2010). Compression was then applied to the RMS intensity using a logarithmic scaling (*x*^0.3^) to model human auditory intensity perception (Stevens, 1955; Biesmans et al., 2016). As mentioned above, this non-linear transformation will improve the efficacy of our linear model without increasing its complexity. The broadband envelope is then obtained by summing over frequency bands. This procedure can be implemented in mTRF-Toolbox using the mTRFenvelope() function (see Box 1). To retrieve the timings of the phonetic features, we use a forced aligner that, given the transcript of the stimulus, will align the onset and offset of each of the phonemes based on the spectrogram (Prosody-aligner, Montreal Forced Aligner) and then map the phoneme timings to their corresponding phonetic features. The resulting phonetic feature time-stamps are represented as a binary matrix (a 1 indicates the occurrence of a phonetic feature), with a different phonetic feature in each column. We will refer to the envelope, spectrogram and phonetic features as **E**, **S**, and **F,** respectively. We also construct a combined acoustic-phonetic representation **FS** by concatenating the features of **S** and **F** (see Figure 2, Speech Representations).

#### Further Considerations

When making decisions about stimulus representations, the resulting number of model parameters relative to the amount of available experimental data should be kept in mind; too many parameters with too few observations can result in the model being overfit to the training data and generalizing poorly to new data, thus it will not be a reliable representation of the system under study. This can be avoided by both reducing the number of model parameters and employing a technique known as regularization (see *Model Training and Testing*).

## Model Design

In addition to choosing the relevant input/output features for our model, there are a number of design considerations that the researcher must make regarding the model itself. For linear models, we do not need to consider the model architecture. Our first design choice is the direction we wish the model to map between the stimulus and the brain, i.e., should it be a forward model or a backward model (see Figure 1C)? The second decision is how much temporal context the model requires: should it map between only single time points or integrate information over windows of several hundred milliseconds? Third, we need to decide whether we want to construct our model using data from one or more subjects in order to enhance model performance and/or generalization. In the following sections, we will discuss the details of each design choice and their use cases. For implementation in mTRF-Toolbox, see Box 2.

**Box 2. Model design implementation.**

Suppose we have a matrix of EEG responses **r**, recorded at a sample rate of *f_*s*_*. To quantify how the EEG responded to changes in stimulus feature **s** over a range of time lags [τ_min_, τ_max_], we construct a forward model. We can use a regularized least squares approach, such as ridge regression, to quantify the forward model weights **w***_*f*_* as follows:

$${\text{w}}_{\text{f}}={\left({\text{S}}^{\text{T}}\text{S}+\text{λ}\text{I}\right)}^{-1}{\text{S}}^{\text{T}}\text{r}$$

where **S** is the design matrix containing the time lagged stimulus features, **I** is the identity matrix and λ is the regularization parameter for controlling overfitting. In practice, we can implement a forward model in one line of code using mTRF-Toolbox by setting the direction parameter Dir to 1 as follows:

model = mTRFtrain(stim,resp,fs,Dir,tmin,tmax,lambda);

The function returns a structure containing the model weights (model.w), the corresponding time lags (model.t) and other model parameters. The design matrix is automatically generated by mTRFtrain() based on the values of tmin and tmax (in milliseconds) and fs (in Hertz). The value chosen for lambda should be validated empirically beforehand using an appropriate cross-validation procedure (for further details, see *Model Training and Testing*). Note, in mTRF-Toolbox, the model weights are normalized by the sampling interval (1/*f_*s*_*) to make them insensitive to the sample rate of the data.

To construct an analogous backward model **w***_*b*_*, we rearrange the equation and apply the time lags to the EEG responses instead to produce the design matrix **R** as follows:

$${\text{w}}_{\text{b}}={\left({\text{R}}^{\text{T}}\text{R}+\text{λ}\text{I}\right)}^{-1}{\text{R}}^{\text{T}}\text{s}$$

Backward models are implemented in mTRF-Toolbox using the exact same line of code as above, but this time by setting the direction parameter Dir to −1. When the backward direction is specified, mTRFtrain() automatically rearranges the equation as above and reverses the time lags to be [−τ_max_, −τ_min_] so that the user does not have to recalculate any of the parameters manually.

As mentioned earlier, the regularization process can act as a low-pass filter, suppressing fast oscillatory components (i.e., noise) in the model. A variant of ridge regression, known as Tikhonov regularization, does this very well and can be implemented in mTRFtrain() by setting the ‘method’ parameter to ‘Tikhonov’.

Additionally, we can implement a series of single-lag models (i.e., models that only map between single time points) by setting the ‘type’ parameter to ‘single’. If tmin and tmax are a range of values (e.g., [0,400]), then mTRFtrain() will return a set of models that map between every single time lag within that range.

### Forward Models

To paraphrase others (e.g., Carandini et al., 2005), the ultimate test of our understanding of sensory processing (and cognitive processing of sensory stimuli) is our ability to predict neural responses to those stimuli. To test hypotheses about those computations and resulting representations, one can build models that attempt to capture how different features of a sensory stimulus affect one’s ability to predict the neural responses (including at different time lags and on different neural recording channels). These models are known as forward or encoding models^4^ and in the EEG literature they are commonly referred to as temporal response functions or TRFs (Ding and Simon, 2012). The weights of a TRF describe how the EEG signal on a given recording channel modulates in response to a unit change in a particular stimulus feature. The temporal dynamics of a TRF typically exhibit a close correspondence to those of an ERP (Lalor et al., 2006, 2009). However, the components of an ERP describe how the EEG signal on a given recording channel modulates in response to the entire stimulus event (that said, ERP researchers often attempt to get at the same information by calculating the difference in ERPs to isolated events that differ only in a specific parameter). Moreover, we can use a TRF to predict the neural response to a given stimulus sequence, and control its smoothness in a way that allows it to generalize well to new data (Haufe et al., 2014; Kriegeskorte and Douglas, 2019). We later discuss ways to ensure confidence in an encoding model’s ability to represent the underlying neural response (see *Interpreting the Model Weights*). Importantly, the interpretability of forward models means that they can be used to both identify processing deficits in particular clinical groups, as well as understand the underlying neurophysiology.

### Backward Models

An alternative to building forward encoding models is to construct a model that maps backward from brain to stimulus (Mesgarani et al., 2009; Ding and Simon, 2012). Such backward models can be used to reconstruct or decode the stimulus features from the neural activity; thus, they are commonly referred to as decoding models or decoders. However, because decoders typically involve optimizing the weighted sum of all neural recording channels simultaneously, the resulting decoder weights are difficult to interpret neurophysiologically (Haufe et al., 2014; Holdgraf et al., 2017). Despite their shortcomings in interpretability, decoders have distinct advantages over encoding models: (1) they do not require pre-selection of neural channels because each channel is weighted according to how informative it is, (2) decoders can utilize independent information on different neural channels to optimally infer stimulus features because they operate on the multichannel neural activity, (3) reconstruction accuracy is usually higher because decoders project to the stimulus domain where we often have direct access to the ground truth (which is not the case for noise-ridden EEG), and (4) decoders can utilize any useful neural information that correlates with the stimulus feature, even if that neural activity did not explicitly encode that feature (Mesgarani et al., 2009). We recommend backward modeling only for reconstructing continuous stimulus features (e.g., speech envelope or spectrogram), and not for reconstructing discrete stimulus representations (e.g., phoneme or word time stamps) which require additional non-linear transformations (but see Huth et al., 2016; Zuk et al., 2019). Forward and backward models thus have distinct uses and can be employed in a complementary manner to investigate both qualitative and quantitative research questions.

### Time Lags

Another important design consideration is the range of time delays or lags to include in our model. We know from the ERP literature that it takes several hundred milliseconds for sensory information to propagate throughout the cortex, with different time lags reflecting different stages of sensory/cognitive processing. Including such time lags in our model allows us to capture the dynamics of the temporal relationship between the stimulus and the neural response, such that the model can utilize relevant information at specific time delays to make better predictions. That means including past stimulus information for making predictions about present neural activity (forward models), and future neural activity for making predictions about present stimulus information (backward models). We recommend using a range of time lags based on the delays at which one expects to see a stimulus-response relationship. This can be determined by referencing previous TRF literature (or even ERP literature), or empirically by systematically evaluating model performance using different lags. For cortical responses, this is typically on the order of hundreds of milliseconds (e.g., 0 to 300 ms), whereas for auditory brainstem responses this is typically on the order of tens of milliseconds (e.g., 0 to 10 ms). For more specific hypotheses, one may wish to limit the time delays to early (0 to 150 ms) versus late (150 to 300 ms), or look at performance as a function of time using a moving window or single lags (see O’Sullivan et al., 2015a; Crosse et al., 2016b). For visualization purposes, it is often desirable to include pre- and post-response lags (e.g., −100 to 400 ms) to illustrate the baseline neural activity or quantify the noise floor. While using a broader range of lags can often yield better predictions, it also increases the number of parameters, which increases susceptibility to overfitting. However, there are techniques to combat overfitting such as regularization (see *Model Training and Testing*).

### Subject Dependency

The last consideration when designing an encoding or decoding model is whether it should be constructed using individual subject data (subject-dependent model) or data from multiple subjects (subject-independent model). Subject-dependent models are more common in the literature because their performance is typically better due to inter-subject variability in the neural responses. However, when it is not possible to collect enough data per subject or per condition, subject-independent models can provide an alternative way of improving model generalization (Di Liberto and Lalor, 2017; Montoya-Martínez et al., 2021). Note, this approach assumes a certain level of homogeneity within each subject group (see *Further Considerations*). There are numerous ways in which to implement subject-independent models:

(a) The model is trained on *n*−1 subjects and tested on the data of the held-out subject,
(b) The model is trained on all *n* subjects and tested on individual held out trials, or
(c) A pre-trained subject-independent model is combined with subject-dependent training data to improve model performance (i.e., transfer learning).

For more information on how to appropriately partition data for training and testing models, see *Model Training and Testing*.

#### Example Experiment

For our example experiment, we chose to go with a forward model design for the following reasons: (1) we wish to compare the neural processing of different stimulus features, hence evaluating model performance in the neural domain makes for a more straightforward comparison (see *Comparing Different Stimulus Features*), (2) we wish to understand any potential group differences in the neural encoding of speech and thus require the model weights to be neurophysiologically interpretable. Based on prior work, we expect to see neural responses to the spectrogram and phonemes over a 300-ms timecourse (Di Liberto et al., 2015; Brodbeck et al., 2018). For visualization and analysis of the model weights, we opt for time lags between –100 and 400 ms in order to observe the entire TRF timecourse, as well as the pre- and post-response activity. For predicting the neural response, we restrict the time lags of the model to between 0 and 300 ms (based on previous empirical testing) in order to optimize its predictive power. Note, we do not merely truncate the temporal features of original model, but rather re-train the model using this restricted range of time lags.

#### Further Considerations

Homogeneity can often be lower in certain clinical populations which may negatively impact the quality of model fit when using a subject-independent design relative to that of the control group. As such, this should be considered when designing the model and deciding between a subject-dependent or -independent design. On the other hand, subject-independent models can potentially be used to empirically demonstrate such differences in homogeneity, if that is of interest to the researcher. For example, if there were no group effects observed using a subject-dependent design, but there were using a subject-independent design, it could suggest increased inter-subject variability in the group with the lower prediction scores.

Furthermore, clinical researchers should consider the possibility of increased within-subject response variability in certain clinical cohorts. For example, the neural response to the same acoustic input could vary over time to a greater extent in certain individuals. This would inevitably impact the quality of model fit for both a subject-dependent and independent design, potentially leading to group differences that do not reflect deficits in the neural processes of interest.

While utilizing as many relevant time lags as possible often leads to better model performance, it can sometimes obscure potential differences in the neural processing between conditions or groups because such differences can manifest within a specific temporal window of the overall neural response timecourse. For example, if our example clinical group differ only in phonetic processing, then it is possible that earlier acoustic processing remains unimpaired, and models based on a broad range of time lags will yield only small group effects or even none at all. Using instead a 2 × 2 design, we can examine model performance based on early versus late lags within each of the groups as well as any potential interactions that may exist. Alternatively, a single-lag analysis could be used to examine model performance as a function of time lag for each of the groups.

## Model Training and Testing

Once the design is in place, the model can be trained and tested on the stimulus-response data. The first step is to partition the data into separate sets to be used for training and testing the model. Then, model hyperparameters are tuned to optimize its ability to predict new data. Lastly, the final model is constructed and tested on held-out data. For implementation in mTRF-Toolbox, see Box 3.

**Box 3. Model training and testing implementation.**

Suppose we want to train a backward model with time lags [τ_min_, τ_max_] to reconstruct particular stimulus features from EEG responses recorded at a sample rate of *f_*s*_*. We must first partition our stimulus and response data into separate training and test sets. In mTRF-Toolbox, the user can partition continuous data into any number of folds and specify one of these folds to be allocated for testing. For example, we can create 10 folds (9 training, 1 test) and specify fold 5 as our test set as follows:

[sTrain,rTrain,sTest,rTest] = mTRFpartition (stim,resp,10,5);

where stim and resp are matrices of continuous data from a single subject. The output variables sTrain and rTrain are returned as 9-by-1 cell arrays containing the training set, and sTest and rTest are matrices containing the test set. Note, if the data were recorded as separate trials and are already stored as cell arrays, this step can be skipped. We then conduct a cross-validation procedure on the training set to identify the optimal value for the regularization parameter λ as follows:

cv = mTRFcrossval(sTrain,rTrain,fs,Dir,tmin,tmax, lambda);

The function returns a structure containing the cross-validation statistics such as the correlation coefficient (cv.r) and the mean squared error (cv.err). Here, the regularization parameter lambda is a vector of values [e.g., 10.^(-6:2:6)] over which we cross-validate our model. We set the direction parameter Dir to -1 to implement a backward mapping. To determine the optimal λ value, we average the performance metrics across folds, and take the λ corresponding to the maximum correlation value (or minimum error value):

[rmax,idx] = max(mean(cv.r));

where rmax is the maximum correlation and idx is the index of the λ value that yielded rmax. Note, if the stimulus features are multivariate, then the researcher will have to decide how to consolidate the data, e.g., take the mean or max across features (see also the banded ridge method in *Comparing Different Stimulus Features*). We then use this lambda value to train our final model as follows:

model = mTRFtrain(sTrain,rTrain,fs,Dir,tmin,tmax, lambda(idx));

where model is a structure containing the relevant model parameters (see Box 2). We then test our model on the held-out test set as follows:

[pred,stats] = mTRFpredict(sTest,rTest,model);

where pred is a matrix of the predicted stimulus features (or EEG responses for a forward model) and stats is a structure containing the test statistics (stats.r, stats.err).

### Data Partitioning

Standard training procedures typically allocate 70–90% of the data to training (training set) and split the rest of the data between validation and testing (validation set and test set). This avoids training and testing on the same data, as this would cause the model to overfit to noise in the dataset, producing a model that would not generalize well to new data. The validation set is used for tuning the model hyperparameters, while the test set is held out until the end and used to evaluate the final model. Note, the allocation of data for testing (i.e., the test set) should be done in an unbiased way at the outset of the analysis, and not re-allocated later in the process based on the outcome of testing (unless there is good reason to do so, such as artifacts or anomalies later identified in the test set).

### Model Training

When training a model, it is crucial that we validate how well our model generalizes at predicting new data. For reliability, it is advisable to obtain multiple measures of model performance using a method such as cross-validation; this is a procedure whereby the training and validation sets are rotated throughout the dataset, allowing us to evaluate the model on every segment of data (except the held-out test set). This is particularly relevant for neural data such as EEG (which are prone to numerous types of sparse artifacts), as we wish to avoid validating our model on a bad segment of data that is not reflective of the entire dataset. If you are working with contiguous time-series data (e.g., EEG), we recommend partitioning the data into shorter contiguous segments for iterative testing. It is typical to partition the data into about 10 folds in order to perform a 10-fold cross-validation (train on 90%, validate on 10%, iterate). If the data are already split into multiple segments or trials (e.g., 20-min × 1-min trials), it can be easier to retain this partitioning and perform a leave-one-out cross-validation (train on *N*−1 trials, validate on 1 trial, iterate). Note, discontinuous segments or trials of data should not be concatenated before model fitting as discontinuities at trial boundaries will introduce noise into our model.

Typically, cross-validation is performed on individual subject data, resulting in a subject-dependent model, but subject-independent models are also useful in the absence of a sufficient amount of data per subject (see *Model Design*). If there are multiple experimental conditions, models must be trained separately on data within each condition, irrespective of whether a subject-dependent or independent approach is adopted. Note, training subject-dependent models on small datasets can be problematic and may require alternative model-fitting strategies which depend on the researcher’s overall goals (see *Fitting Models to Small Datasets* in *What Can Go Wrong?*).

### Regularization

It is important to ensure that our model does not overfit to the noise in our training data, especially when there is a limited amount of data or the model has a large number of parameters. This can be achieved by employing a technique called regularization. Regularization of linear models can be implemented in a number of different ways, most of which converge on the same solution and yield similar performance (see Wong et al., 2018). A common method known as ridge regression uses a hyperparameter called the ridge parameter to control the correlations between the weights in the model. This method enforces a smoothing on the model weights by penalizing those with large values (i.e., the square of the weights), reducing the variance and producing a model that generalizes better to new data. A variant of ridge regression, known as Tikhonov regularization, imposes a constraint on the first derivative of the model weights which provides temporal smoothing and dampens fast oscillatory components in the solution (Lalor et al., 2006; Crosse et al., 2016a). However, this approach may cause cross-channel leakage for multivariate input features (e.g., spectrograms, phonemes etc.), in which case it may be better to use standard ridge regression (both methods are provided as options in mTRF-Toolbox). Another common method, known as LASSO (and its variants), controls the number of weights with non-zero values (Tibshirani, 2011).

In practice, regularization takes care of two important issues during model fitting: (1) if a model has many parameters relative to observations and is thus susceptible to overfitting, it reduces the amount of non-zero weights or their absolute value to produce a model that generalizes better, (2) neighboring datapoints within both the stimulus and EEG can often be highly correlated along multiple dimensions (e.g., space, time, frequency), also known as collinearity, leading to smearing or leakage along such dimensions as the model fitting attempts to adjust for highly correlated neighboring samples (see Figure 7E in Crosse et al., 2016a). Because linear regression can be implemented using an efficient closed-form solution (i.e., the Normal Equation), the model parameters themselves do not need to be tuned using an iterative optimization algorithm such as gradient descent. Thus, tuning the regularization parameter is often the most important part of training your linear model and should be done with great care (see also *What Can Go Wrong?*).

### Model Testing

To evaluate model performance, the model is first used to predict a set of output features from a held-out set of input features (i.e., the test set). For a forward model, we predict a set of unseen neural responses and for a backward model we reconstruct a set of unseen stimulus features. For subject-dependent models, the test set is typically a single segment or trial of data from that same subject. For subject-independent models, the test set can be either (1) multiple segments/trials of data from a held-out subject, or (2) an individual segment/trial of data from a subject whose remaining trials are included in the training set (see Subject Dependency section in *Model Design*). The latter approach will likely yield better performance due to the inclusion of subject-dependent data in the model.

We then evaluate model performance by calculating a predictive score based on the similarity (or error) between our prediction and the original signal (i.e., ground truth). It is common to use Pearson’s correlation coefficient as a measure of similarity because it quantifies how linearly close the dynamics of the prediction are to the ground truth, irrespective of the magnitude or mean of the signal being predicted. In the event that the relationship between the predicted and actual signals is not linear, a Spearman’s correlation can instead be used. It is also common to measure the error (i.e., the absolute distance) between the predicted and actual signals. Standard error metrics include the mean squared error or mean absolute error which, unlike correlations measures, rely on the absolute magnitude of the signals. All of the above metrics can be implemented using mTRF-Toolbox (see Box 3).

#### Example Experiment

To build our encoding models, we first have to partition our data into our training and test sets. We opt to allocate 80% of the data to training, and 20% to testing (i.e., 12 training trials and 3 test trials). Each subject’s test set is taken from the middle of the recording session to ensure good quality EEG data (i.e., the electrodes are well settled by then and the subject is likely not too fatigued). Because we opted to use ridge regression to train our models, we must tune the regularization (ridge) parameter in order to avoid overfitting our model to the data. We iteratively train and validate our models using a leave-one-out cross-validation procedure on the training set to identify the optimal regularization value (12 training trials equates to a 12-fold cross-validation). We carry out this procedure separately for any stimulus feature models we intend to evaluate and compare (i.e., **F**, **S**, and **FS**), as it is likely that each representation will require a different amount of regularization. Once the ridge parameter is tuned, we train our final model on the entire training set and test it on our held-out test set. In the absence of any strong hypothesis about lateralization (Hickok and Poeppel, 2007), we average prediction accuracies across 6 bilateral auditory-responsive EEG channels (12 in total) in order to evaluate model performance.

#### Further Considerations

If using a leave-one-out cross-validation approach, consider how many trials or segments of data are going into the cross-validation. For example, if there are 10 trials, then leave-one-out performs a 10-fold cross-validation (trains on 90%, validates on 10%). However, if there are many more than 10 folds (e.g., 50 folds), then the cross-validation procedure may become biased because there will be very little variance in the training sets across iterations (each training set only differs by a mere 2% for 50 folds). Furthermore, this increases the risk of the validation set becoming highly correlated with the training set, which would also bias the result. On the other hand, if there are too few folds (e.g., 2 folds), this will significantly reduce the amount of data in each training set (each training set only accounts for 50% of the data), thus potentially resulting in vastly different result across folds. For this reason, it is usual to use around 10 folds.

Memory usage issues may arise if you are training your model on very large segments or trials of data. When performing cross-validation with mTRF-Toolbox, if the trials used are too long (e.g., 5-min trials), you can use the ‘split’ parameter in mTRFcrossval() to partition them into smaller segments in order to reduce memory usage (see also the ‘fast’ parameter for efficient memory usage). While splitting the trials into smaller segments results in a greater number of cross-validation folds, it does not greatly increase computation time because of the efficient cross-validation procedure used in mTRFcrossval()^5^.

Instead of evaluating the model over the entire fold we can use a smaller window size (e.g., 20 s) to compute the correlation and error in order to obtain multiple prediction scores per fold. In mTRF-Toolbox, the window size can be specified via the ‘window’ parameter in mTRFcrossval() and mTRFpredict(). Note, reducing the window size below a certain threshold (typically < 10 s) will begin to reduce the average correlation value and also result in spuriously high and low estimates. However, this feature may be useful for researchers interested in real-time decoding applications such as brain-computer interfaces (BCIs), and such spurious correlation estimates can be managed over time by using Bayesian filtering techniques such as fixed-lag state-space models (e.g., Miran et al., 2018).

## Evaluating Model Integrity

While the use of encoding and decoding models is well established in cognitive neuroscience and has been shown to reliably quantify different sensory and cognitive processes, it is still paramount that we assess the integrity of each individual participant’s data and resulting model before proceeding with our analysis. There are numerous factors that could lead to the construction of a model that does not reflect a meaningful stimulus-response relationship: (1) excessive noise in the neural recording, caused by movement or external interference (see *Data Preprocessing*), such that it obscures the stimulus-related part of the response, (2) poor subject compliance due to lack of motivation or fatigue, resulting in the neural recording reflecting little stimulus-relevant information, (3) certain subjects can exhibit inherently weak responses on the scalp to certain stimuli due to various anatomical reasons. Thus, it is critical to establish from the outset that a given participants’ model is meaningful and performing well above chance level. Here, we describe how to compute chance level for linear models and quantify their significance. For implementation in mTRF-Toolbox, see Box 4.

**Box 4. Model evaluation implementation.**

Regression models are typically evaluated using correlation or error metrics. In mTRF-Toolbox, we can compute such metrics for a forward or backward model as follows:

[r,err] = mTRFevaluate(y,pred);

where y is the ground truth and pred is the model prediction. The function returns two evaluation metrics: r is the Pearson correlation coefficient and err is the mean squared error (MSE). Alternatively, we can specify a Spearman correlation using the ‘corr’ parameter and the mean absolute error (MAE) using the ‘error’ parameter. The mTRFevaluate() function is automatically called by other functions in mTRF-Toolbox such as mTRFpredict() and mTRFcrossval() for computing such performance metrics.

In order to evaluate whether such performance metrics are statistically significant, we can use a permutation-based approach which cross-validates models using mismatched permutations of the stimulus features and neural responses. In mTRF-Toolbox, this can be implemented as follows:

stats = mTRFpermute(stim,resp,fs,Dir,tmin, tmax,lambda);

The function returns a structure containing the usual cross-validation statistics such as the correlation coefficient (stats.r) and the mean squared error (stats.err). Optionally, the ‘nperm’ parameter can be used to specify the number of permutations to perform.

### Establishing Data Integrity

We have previously described our plan to construct a forward model in order to quantify the neural tracking of acoustic and phonetic features. But is the signal-to-noise ratio (SNR) of our EEG data good enough to address such research questions? Before constructing our forward model, and as a first pass to assess whether the EEG responded to changes in the stimulus features of interest, we recommend first using a backward model to reconstruct a simple, continuously time-varying stimulus feature that is known to be reliably tracked by the EEG signal. For example, we could use the envelope of the audio recording, as it serves as a good approximation of the perceived stimulus intensity. The reason we recommend this initial backward modeling step is that it is better able to detect the presence of neural tracking of the stimulus than forward modeling. As previously mentioned, backward modeling has the distinct advantage of utilizing all the available EEG channels simultaneously, as well as projecting to the stimulus domain where we typically have direct access to the ground truth (unlike noise-ridden EEG). If the accuracy of the resulting reconstruction is above chance, then we can then confirm that the EEG contains genuine information relating to the desired stimulus feature.

### Defining the Null Distribution

Quantifying chance-level performance of encoding and decoding models is non-trivial. For any statistical test, defining the null distribution makes inherent assumptions about the distribution of the data. Thus far, we have suggested validating the model prediction using Pearson’s correlation coefficient, but unfortunately the standard statistical tests for correlation are not appropriate to determine if neural tracking is above chance. This is because they usually assume independent samples, but EEG data and naturalistic stimuli will typically exhibit a high correlation between neighboring time points or channels/features meaning adjacent samples are not independent. Instead, we recommend computing a null distribution of prediction accuracies using randomly shuffled permutations of the data at hand (Nichols and Holmes, 2002; Combrisson and Jerbi, 2015). This process involves random pairings of EEG responses to stimulus representations (for example, by shuffling the trial label). Note, however, that significance testing using this null distribution is only as sensitive as the number of trials. For example, 10 trials will produce at most 100 pairings, and randomly selecting 1000 pairings will produce several repeated pairings.

Alternatively, if the trials are sufficiently long and if there were not enough trials to get a null distribution of random pairings, one can (randomly) circularly time shift the stimulus relative to the reconstruction. This maintains the temporally correlated structure of both the stimulus and the reconstruction, while eliminating the phase relationship between the stimulus and reconstruction (Bialek et al., 2005). One downside of this approach is that it does not account for time-locked responses that are present in all trials (such as an evoked response at the start of the trial) and could produce a false positive if the uniqueness of the reconstruction is of interest. Additionally, discontinuities introduced by circular shifting (if the start and end points of the signal are very different) can produce inappropriate null distributions (Harris, 2020), but this issue is somewhat minimized by high-pass filtering the data (see *Data Preprocessing*). The experimenter should decide on the most appropriate method for creating a null distribution, based on the experiment design and the number and duration of the stimuli.

### Quantifying Significance

Once a null distribution has been defined, we can then calculate a *p*-value for an individual participant’s model by averaging their performance measures (e.g., correlation coefficients) across trials and quantifying the proportion of the null distribution that falls above their average performance value (i.e., a one-tailed test). Alternatively, to account for the variability in true correlations, we can compute a measure of sensitivity known as d-prime (*d′*) as follows:


$$d^{'}=\frac{\mu_{\text{true}}-\mu_{\text{null}}}{\sqrt{\frac{1}{2}\left(\sigma^{2}{}_{\text{true}}+\sigma^{2}{}_{\text{null}}\right)}}$$

Where *μ* is the mean correlation and σ is the standard deviation. To calculate significance for a group of participants, we compare the sample of null correlations to a sample of true correlations using an appropriate means test.

#### Example Experiment

Before comparing speech processing in our clinical and control groups, we wish to validate the general integrity of our data, i.e., whether we can reliably detect neural tracking of the stimulus in the EEG data at all (see also *Further Considerations*). For reliability, we construct a backward model that maps from the 128-channel EEG to the speech envelope, using the same time lags selected for our forward model (0–300 ms). We test our model on held-out data to compute the average prediction score and evaluate its significance against a null distribution derived from random permutations of the data (see Box 4). Having established that the average prediction score is above the 95th percentile of the null distribution, we conclude that the stimulus was reliably encoded in the EEG data and move forward with our main analysis.

Next, we have chosen to evaluate our forward encoding models based on the average prediction score at 6 bilateral auditory-responsive EEG electrodes (12 in total). We evaluate the models by averaging their prediction accuracies and perform the same permutation procedure on that average, randomly shuffling trials to generate a null distribution. Because all three models (**S**, **F**, and **FS**) perform better than chance, we conclude that acoustic and phonetic features were reliably encoded in the EEG data. However, we know that acoustic and phonetic features are highly correlated in speech. Next, we will disentangle the relative contributions of these different stimulus features.

#### Further Considerations

In addition to running an initial backward model analysis to assess data integrity, one could also include a short ERP study before the main experiment. This could consist of very simple stimuli such as flashes or beeps, depending on the modality of interest. While not a direct measure of continuous stimulus tracking, it would give the researcher some idea of the quality of their data independent of the modeling analysis which, when performed incorrectly, can produce poor results despite the data potentially being of good quality. Another advantage to this approach is that there are ways to rapidly analyze ERPs online and establish data quality early on in the experiment. This can help in deciding whether to continue running the entire study on a given participant or terminating it early to save you both time.

## Comparing Different Stimulus Features

One of the strengths of using linear models is the ability to define multiple stimulus representations, such that we can target specific stages of processing along the sensory/cognitive pathway. While we can directly compare the performance of various models constructed using the different stimulus features, it is likely that such features contain overlapping information along one or more dimensions leading to redundancy between the corresponding models. However, it may be necessary to quantify the unique contribution of a specific feature if the researcher is interested in obtaining a measure of something like speech-specific processing. There are multiple ways of approaching this problem. For implementation in mTRF-Toolbox, see Box 5.

**Box 5. Comparing stimulus features implementation.**

In order to compare between the unique contributions of different stimulus features to the observed brain activity, we must account for statistical and perceptual redundancies between those features. As outlined in this section, one way to do this is to compute the difference in prediction score between combined and individual feature models. A more direct method involves partialing out the contribution of one set of features from the EEG data, then modeling the relationship between another set of features and the residual EEG. In mTRF-Toolbox, we can implement this partialing procedure and obtain the residual EEG responses as follows:

resid = mTRFpartial(stim,resp,fs,Dir,tmin,tmax, lambda);

As discussed in this section, we have adapted banded ridge regression as described in Nunez-Elizalde et al. (2019). In short, this function performs leave-one-out cross-validation while applying different levels of regularization separately to “bands” of stimulus features. Suppose we wish to apply 2 bands of regularization to a stimulus representation that has 10 features, with 5 features per band. First, we define the range of regularization values for each band and how we wish to group the features in each band:

band1 = 10.^(-6:2:6);
band2 = 10.^(0:2:12);
lambda = [band1;band2];
grouping = [1,1,1,1,1,2,2,2,2,2];

Then we perform our banded regularization in mTRF-Toolbox as follows:

cv = mTRFcvbanded(stim,resp,fs,Dir,tmin,tmax,lambda, grouping);

The function returns a structure containing the usual cross-validation statistics. It is important to consider that the computation time grows exponentially with the number of bands in an exhaustive search $\left(N_{\lambda }^{N_{bands}}\right)$. However, the cross-validation procedure can be optimized via the search algorithm in MATLAB, which is implemented in the following mTRF function:

cv = mTRFcvsearch(stim,resp,fs,Dir,tmin,tmax);

Note that lambda is no longer a required input. Instead, there is an optional parameter, ‘init,’ which specifies an initial lambda value for each band (default value is 1). Additionally, if the ‘grouping’ parameter is included, the function performs the search using banded ridge regression.

### Combining Stimulus Features

This approach first quantifies the combined contribution of multiple features by concatenating them together along the columns of the design matrix and constructing a “combined” model (see Figure 2, Speech Representations; Di Liberto et al., 2015; O’Sullivan et al., 2017; Desai et al., 2021). To evaluate whether an individual stimulus feature contributes unique information, separate models are fit using the individual features and the differences in prediction scores between the combined model and the individual models are computed. While this is an indirect way to quantify such unique contributions, it has been shown to be predictive of behavior (Di Liberto et al., 2018a). Note, when combining multiple stimulus features in a single matrix, regularization may not affect the features equally, especially if the frequency content differs between the features (see *Further Considerations*). In general, this method is more suited to forward modeling. Comparing the neural processing of different stimulus features in the neural domain (i.e., forward modeling) is straightforward because the output of the models is similar (i.e., continuous neural responses). Comparing them in the stimulus domain (i.e., backward modeling) is complicated by the fact that some representations might be continuous (e.g., the speech envelope) and others might be discrete (e.g., sparse binary representations such as phoneme or word onset times), requiring additional non-linear transformations. Thus, reconstruction of such non-continuous categorical features may introduce statistical errors that are not reflective of the underlying neural processes.

### Partialing Out Contributions

A more direct way to deal with common brain activations produced by redundant stimulus features is to partial them out of the neural data (O’Sullivan et al., 2021). The residual neural signal can then be mapped in a forward or backward direction to the other stimulus features (i.e., the features that were not regressed out of the neural data) in order to define a model that captures the neural dynamics unique to those features. Vice versa, the same procedure can then be implemented for the other set of features. Depending on how much overlap there is between the two sets of features (temporal, spectral etc.), the residual neural signal may contain considerably less information about the other stimulus set after the first has been partialed out, resulting in very low prediction scores. However, when comparing different subject groups or experimental conditions, we are often more interested in the prediction score relative to that of another group or condition.

#### Example Experiment

**FIGURE 3:**
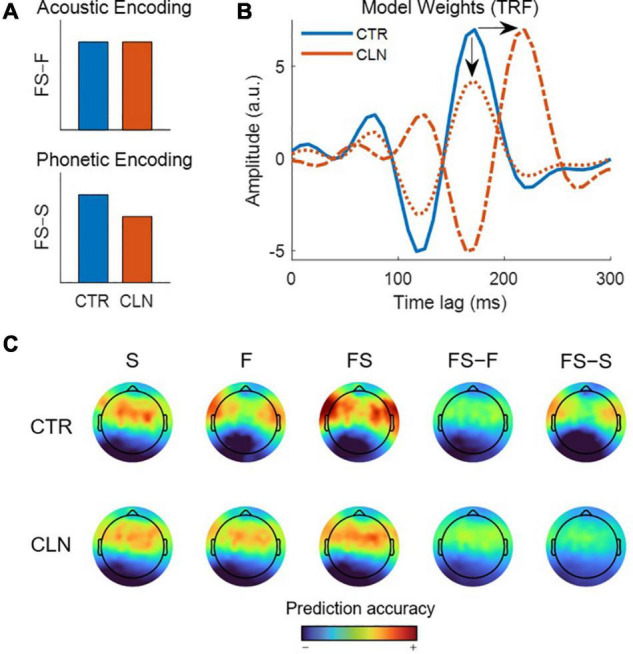
Hypothetical results for example experiment using forward models. **(A)** We hypothesize that there is a deficit in the neural processing of phonetic features in a clinical group (CLN, red) relative to our control group (CTR, blue). We obtain a measure of acoustic encoding by quantifying the differential in prediction scores (e.g., correlation coefficient) between the combined spectrogram + phonetic features model (FS) and the individual phonetic features model (F). Similarly, we obtain a measure of phonetic encoding by quantifying the differential in prediction scores between FS and the individual spectrogram model (S). We would expect to see no group effect in acoustic encoding, whereas for phonetic encoding, we would expect to see reduced performance in the clinical group, indicative of a deficit in phonological encoding. **(B)** Hypothetical group differences in average phoneme TRF weights. The group average TRF for control subjects is shown as the blue trace. One possible scenario shows a reduction in TRF amplitude for the clinical group (red dotted trace), which could indicate either reduced neural activity or increased inter-subject variability. Alternatively, there could be a difference in TRF latency (red dashed trace), due to delayed neural processing. **(C)** Hypothetical scalp topographies of forward model prediction scores for control and clinical groups. These hypothetical results depict differences in prediction score, as per our hypothesis, but also differences in the distributions of those scores across the scalp.

Our original hypothesis was that we would observe group differences in phonological but not acoustic processing. We have already determined how well we can predict the neural responses using the individual and combined feature representations, **S**, **F**, and **FS** (see *Example Experiment* section in *Evaluating Model Integrity*). By subtracting the **S** prediction scores from the **FS** prediction scores (**FS**−**S**), we can quantify how much the **FS** predictions were improved by the inclusion of phonetic information, in addition to the acoustic information. Similarly, we can isolate a measure of acoustic processing by quantifying the difference in prediction scores between our combined model and phonetic model (**FS**−**F**). The results of our hypothetical experiment are illustrated in Figure 3 and show that acoustic processing (**FS**–**F**) is similar for both groups, but that phonemic processing (**FS**–**S**) is reduced in our clinical group, consistent with our hypothesis that there is a phonological-specific impairment in our clinical group.

#### Further Considerations

Standard regularization methods, such as ridge regression, use a single regularization parameter, even for multivariate and combined stimulus representations. This may lead to sub-optimal model fits and poor predictions because they apply the same level of regularization across all feature types (e.g., spectrogram, phonetic features). The amount of regularization required for each feature can vary depending on several factors such as frequency content, magnitude, sparsity, and SNR. In the example combined model described above, we have two markedly different types of speech features; the spectrogram is represented as a continuous variable across 32 frequency bands, while the phonetic features are discrete, binary variables across 19 phonetic categories. A solution here is to apply separate levels of regularization to specific bands of features, one band for the acoustic features and one for the phonetic features, a technique known as *banded ridge regression* (see Box 5). This approach has the added benefit of reducing spurious correlations between features. Fit separately, single-feature models may produce prediction accuracies that are over-estimated due to correlations between the features. Banded ridge regression can reduce this problem by “decorrelating the model features to an amount determined by their covariance and the regularization parameters” (Nunez-Elizalde et al., 2019). Individual feature models can then be evaluated separately using the weights derived for the combined model via banded ridge (for further details, see Nunez-Elizalde et al., 2019).

## Interpreting the Model Weights

One of the major advantages of using linear models is that their weights are easy to interpret, a property that is highly desirable when studying physiological systems. In this section, we discuss interpretation in the context of forward and backward models, model generalization, as well as presenting simulations that demonstrate the impact of data quantity and SNR on prediction score.

### Forward Models

**FIGURE 4:**
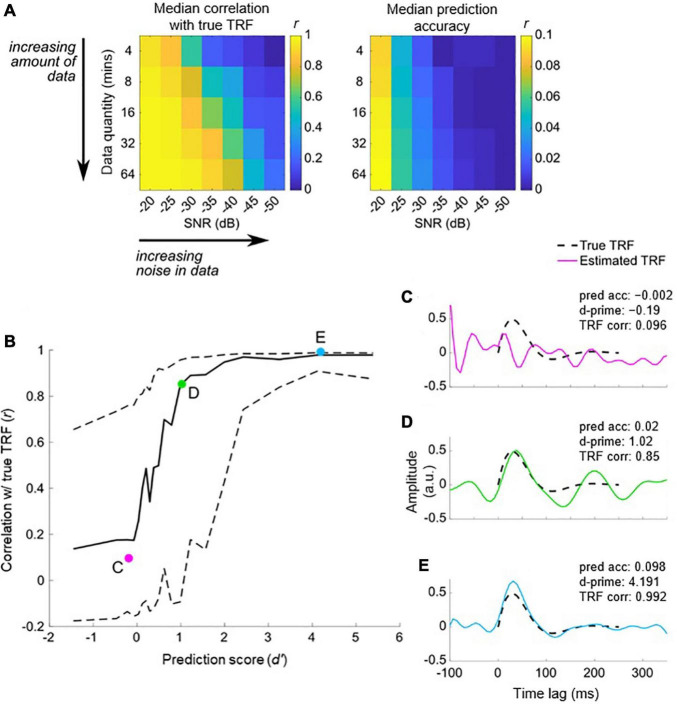
Simulation of model performance as a function of data quality and quantity. Neural data were simulated using a TRF-like response with EEG-shaped noise (both filtered between 2–15 Hz) and randomly generated stimuli at different SNRs in the range [−20, −50] dB and different numbers of trials (each trial is 1 min long). Each pairing of SNR and amount of data was simulated 100 times. **(A)** Median correlation coefficient between the true and modeled TRF (left) and median prediction accuracy (right) as a function of data quantity and SNR. Leave-one-trial-out procedure was used to quantify prediction accuracy of the trials, and for each simulation we averaged prediction accuracies across trials. Both prediction accuracy and the model estimate of the true TRF decrease with increasing amount of noise and decreasing number of trials. In light of this, we collapsed the data across conditions and plotted the relationship between prediction accuracy and model TRF to true TRF correlation across simulations **(B)**. d-prime prediction accuracy was used to normalize for differences in the null distribution, which can vary with the frequency range of the data. Shown for each condition are the median (solid line) and the 10–90% quantiles (dashed lines). As prediction accuracy decreases, the model estimate of the true TRF gets less reliable. **(C–E)** Shown are example stimulations with poor, moderate, and good estimates of the TRF, respectively (**C:** −45 dB SNR, 64 min; **D:** −25 dB SNR, 4 min; **E:** −20 dB SNR, 64 min). The root-mean-square of the estimated TRFs were normalized in this plot to match the true TRF. The d-primes and correlations between the true and predicted model for each simulation have also been labeled in **(B)** using the same colors of the traces in **(C–E)**.

**FIGURE 5:**
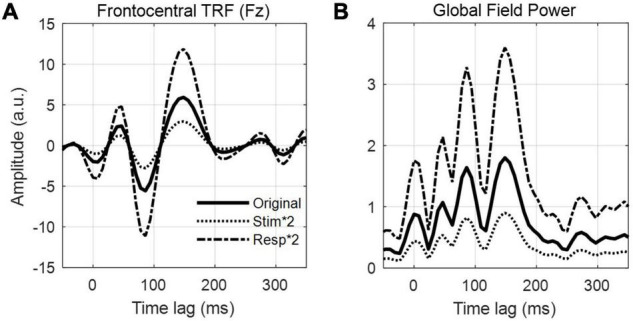
Effects of stimulus-response normalization on TRF amplitude. **(A)** Frontocentral TRF (channel Fz) calculated from 15 min of speech-EEG data using the envelope feature. The plot shows the TRFs calculated using the original, unnormalized data (bold trace), the stimulus features scaled by a factor of 2 (dotted trace) and the neural response scaled by a factor of 2 (dashed trace). **(B)** The global field power – calculated as the standard deviation across all 128 EEG channels – for the same 3 conditions as in panel **(A)**.

The analysis and interpretation of forward model weights are similar to that of an ERP, although a few caveats should be noted. Firstly, a forward model is not an ERP because its weights are fitted to optimally predict the neural response at a given sensor, thus, the relationship between the weights is mathematically relevant by design (Kriegeskorte and Douglas, 2019). Forward models trained on naturalistic stimuli produce convincing representations of neural responses with similarities to ERPs (Figure 3B; Lalor et al., 2006, 2009), but correlations between stimulus parameters can affect this interpretation. Secondly, if our goal is to capture the underlying neural response using linear modeling, we have to ensure that the model generalizes well at predicting new data. Both the model’s approximation of the underlying response and its performance vary as a function of SNR and the amount of available data (see Figure 4 and *Further Considerations*). As such, it is crucial to evaluate the model’s predictive power prior to interpreting the model weights. It can be tempting to interpret specific temporal or spatial patterns in the model weights as reflecting something interesting about the underlying brain activity, particularly when they appear to satisfy our preconceived notions or hypotheses regarding brain function. However, if a model has no predictive power, then it is likely overfit to noise in the data. When quantitatively comparing forward model weights between different conditions or groups, the researcher must be particularly mindful of the normalization procedures applied to the stimulus and response features during the preprocessing stage. Any slight differences in normalization could impact the model weights and masquerade as underlying neural effects (see Figure 5 and *Further Considerations*).

### Backward Models

While it is possible to quantitatively compare patterns of backward model weights across multiple experimental conditions (e.g., Crosse et al., 2015), it is not recommended to interpret such patterns in a qualitative manner or through the lens of neurophysiology. As previously stated, the weights corresponding to backward models are not physiologically relevant because they map in the acausal direction, i.e., in reverse to the natural flow of information of the system under study. However, there are transformations that can be applied to backward models in order to observe the corresponding forward representation (see Haufe et al., 2014). In mTRF-Toolbox, the function mTRFtransform() allows the researcher to perform this transformation in one line of code (see Box 6). We strongly recommend using the resulting transformed model merely for the purpose of interpretation, and not for predicting neural responses to novel stimuli. There is no guarantee that the resulting forward model from the transformation is sufficiently regularized and optimal for neural prediction.

**Box 6. Interpreting model weights implementation.**

Suppose we want to train a backward model with time lags [τ_min_, τ_max_] to reconstruct particular stimulus features from EEG responses recorded at a sample rate of *f_s*. We would first optimize the regularization parameter λ using an appropriate cross-validation procedure as outlined in Box 3. Once complete, we can train our backward model in mTRF-Toolbox as follows:

bmodel = mTRFtrain(stim,resp,fs,Dir,tmin,tmax,lambda);

Aside from using our backward model for decoding purposes, we may wish to gain insight into the underlying neurophysiology. While we cannot directly interpret the weights of our backward model in its current form neurophysiologically, we can transform it into the corresponding forward model as described in Haufe et al. (2014) using mTRF-Toolbox:

fmodel = mTRFtransform(bmodel,resp);

The function returns a structure fmodel containing the same model parameters as those returned by mTRFtrain(), except for the bias term. Thus, the resulting model cannot (and should not) be used for prediction. For that, we recommend directly constructing an optimized forward model. We can however analyze the weights of our transformed forward model as per usual. The model weights can easily be plotted as a function of time and/or features using mTRF-Toolbox:

h = mTRFplot(model,type,feat,chan);

The function takes the entire model structure and plots the weights of the specified features and channels. The type parameter can be used to specify a univariate TRF plot (‘trf’), a multivariate TRF plot (‘mtrf’) or a global field power plot (‘gfp’), i.e., the standard deviation across all neural channels (see Figure 5).

### TRF Simulations

To demonstrate this issue of model performance and interpretability as a function of SNR, we conducted a series of linear modeling analyses on simulated EEG data with a frequency range relevant to speech tracking (2–15 Hz, roughly theta to beta). For each simulation, we: (1) randomly generated a time-varying ‘stimulus’ in this frequency range and convolved it with the expected TRF, (2) added noise with approximately the same spectrum as EEG, and (3) trained and tested the model using a leave-one-trial-out procedure, where each trial was 1 min long. Each simulation was run 100 times. Based on these simulations, we show that model performance improves with more data and when the data are less noisy (i.e., higher SNR), and thus the resulting model has a high correlation with the true response (Figure 4A). As the amount of data or the SNR decreases, both model performance and model fit drop. The ‘noise’ in these simulations refers to any component that does not track the stimulus. Practically, this includes external mechanical and electrical noise as well as uncontrollable factors such as neural activity from other brain regions and processes that are not of interest (see *Data Preprocessing*).

Collapsing the results across SNR and data quantity, we see a direct relationship between the reliability of the model’s representation of the true response and the prediction score (Figure 4B). Here, we use a d-prime measure, which is quantified relative to a null distribution produced by randomly circularly shifting the trials (see *Evaluating Model Integrity).* When the prediction score is low, the model is a poor representation of the true response. More specifically, the correlation between the predicted and true model plateaus for d-prime prediction scores around 2, and the model is often (>90% of the time) a reliable representation of the true response (Figures 4B–E for example simulations). Thus, we strongly recommend evaluating the model’s predictive power prior to interpreting the model weights.

#### Example Experiment

So far, we have compared the control and clinical groups based on their model prediction scores (see *Comparing Different Stimulus Features*). Differences in prediction scores are driven by differences in the underlying neural responses to the stimulus, which will be captured by the model weights themselves. Such differences might manifest as a change in magnitude, latency or spatial distribution of the TRF components (see Figure 3B). For simplicity, we will focus on a univariate stimulus feature, but this approach can be extended to examine multivariate stimulus features.

To study the TRF weights in our example, we carried out a two-step procedure. First, we identified time lags where the TRF weights were significantly different from zero within each group. We used paired, two-tailed permutation tests based on the *t*-statistic with *t*_*max*_ correction via PERMUTOOLS^6^, but this analysis could also be conducted, for example, using a cluster mass statistics method (Maris and Oostenveld, 2007). Similar statistical methods were used to evaluate between-group effects (i.e., unpaired permutation tests). There was no statistical evidence to suggest group differences in the acoustic model weights, whereas in the phonetic model there were significant group differences at time lags of ∼200 ms.

An alternative data-driven approach consists of performing a cluster analysis on the TRF weights to identify clusters with significant TRF components. While a single 2D cluster analysis (EEG channel × time lag) is sufficient for univariate inputs, multiple 2D cluster analyses should be run for multivariate inputs. The analysis can then be carried out for each cluster by averaging each set of electrodes or by focusing on the centroid channel of each cluster.

#### Further Considerations

Even for experimental setups with low electrical noise and well-behaved subjects, the SNR of the recorded neural activity can vary across subjects due to differences in cortical folding, anatomical spatial filtering or the relative activity in other brain regions that are not of interest. When the neural data are particularly noisy, it can result in poor TRF estimates, even with a large amount of data (see Figures 4B,C). This must be taken into consideration when analyzing model weights across a large cohort of subjects, particularly when defining temporal or spectral windows to quantify specific TRF components.

When comparing the magnitude of TRF weights between different conditions or groups, be very mindful of how the data were normalized (if any normalization procedures were used in the preprocessing stage). Specifically, increases in the stimulus feature values will result in a reduction in TRF amplitude, and increases in the neural response values will result in an increase in the TRF amplitude (see Figure 5). The relationship is linear and will act in the opposite direction for a reduction in the stimulus or response values. Thus, we suggest avoiding normalizing the neural data altogether, unless using a common factor for all datasets, such as the amplifier microvolts/bits conversion ratio. Similarly, care must be taken when normalizing the stimulus features, particularly if different stimuli are used across conditions or subjects. Again, using a common normalization factor can avoid biasing particular conditions or subjects.

Depending on the algorithm used to model the stimulus-response relationship, the sample rate of the data can also affect the magnitude of the model weights. In mTRF-Toolbox, the model weights are insensitive to the sample rate because the algorithm normalizes the weights by the sampling interval (1/*f*_*s*_). This means the user can run multiple analyses at different sample rates without introducing inconsistencies in the magnitude of the model weights and compare the amplitude of TRF components calculated at different sample rates.

## What Can Go Wrong?

### Fitting Models to Small Datasets

The first step of fitting a stimulus-response model is to tune any hyperparameters, such as the regularization parameter (*see Model Training and Testing*). A common approach is to do an exhaustive parameter search over a specific range of values. While an exhaustive search can be costly when there are multiple hyperparameters to tune, it can be feasible in the case of a single hyperparameter, such as the regularization (ridge) parameter in ridge regression. The ridge parameter can be tuned such that it optimizes the model prediction score, such as the correlation or MSE between the predicted and actual neural response.

**FIGURE 6:**
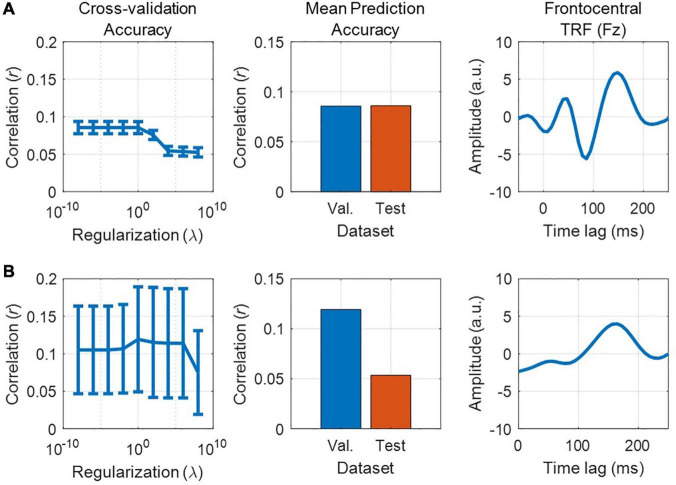
Fitting models to small datasets. **(A)** Forward model (TRF) trained on 15 min of speech-EEG data using the speech envelope feature. Left, mean cross-validation accuracy (5-fold) for frontocentral channel Fz. Error bars indicate SEM across folds. Middle, mean prediction accuracy (Pearson’s *r*) at Fz for validation and test sets using optimized regularization parameter. Right, optimized TRF weights at Fz. **(B)** Forward model trained on only 30 s of the same dataset. Left, mean cross-validation accuracy (5-fold) at Fz. Middle, mean prediction accuracy at Fz for validation and test sets using optimized regularization parameter. Right, optimized TRF weights at Fz.

The optimal level of regularization may differ across subjects (and sessions), as it depends on factors such as the SNR of the neural data. For this reason, hyperparameter tuning is usually performed on individual subjects. However, this approach can be problematic when working with small datasets. Consider, for example, an EEG dataset with 30 participants, each with less than 1 min of data per condition. It is possible that such a small amount of data is not sufficient to fit models that generalize well to new EEG data or whose weights provide meaningful neurophysiological insight. This issue may prevent us from deriving useful models at the individual-subject level using an exhaustive search. Figure 6 illustrates how hyperparameter tuning via cross-validation at the individual-subject level yields neurophysiologically plausible TRF weights when there is a sufficient amount of data (i.e., 15 min of data, Figure 6A), but fails to do so when we limit training to a smaller, insufficient amount of data (i.e., 30 s of data, Figure 6B). Instead, the cross-validation procedure overestimates the amount of regularization in order to shrink the weights and reduce their contribution to the prediction, leading to a grossly underfit model with little resemblance to a typical auditory cortical response. Note, model performance appears to be improved during cross-validation for the smaller dataset, but performs worse on the test set which is ultimately how we should evaluate the quality of the model. One possible solution is to identify a suitable denoising procedure to increase the SNR of the neural data (see step 6 in *Data Preprocessing*). If that is not possible or sufficient, it may be possible to improve the model fit by including additional assumptions. For instance, if we assume the neural responses for individuals within the same group are relatively consistent, data from all participants can be pooled together to determine a unique regularization parameter. This could be implemented in different ways that depend on particular assumptions and the dataset, e.g., should the neural data be normalized before it is pooled? How are the data pooled? See *Subject Dependency* in *Model Design*. The researcher may then decide to use the resulting regularization parameter to study the TRFs at the group level or to restrict hyperparameter tuning at the individual-subject level. Alternatively, a fixed value of regularization could be used across subjects^7^. This constant could be determined based on values used in relevant previous studies, or empirically based on a larger, more reliable dataset with similar statistics. It could also be determined empirically based on the characteristics of the group-average TRF estimate, as the lowest value such that any increase would result in no visible improvement in the estimate (see Lalor et al., 2006). While the latter approach is less autonomous than standard machine learning validation procedures – requiring the researcher to draw on the relevant literature as well as their own expertise in neurophysiology – it may in certain circumstances be an optimal solution.

### Poor Hyperparameter Tuning

Consider the scenario where a researcher wants to assess auditory processing in a particular cohort using a music listening paradigm. Before doing so, they plan to first validate their analysis pipeline on a publicly available dataset described in Di Liberto et al. (2020). In this previous study, 64-channel EEG data were recorded in 20 participants while they listened to ten monophonic piano melodies by J.S. Bach, each occurring three times, and presented in random order. The researcher intends to calculate TRFs for each participant using the temporal envelope of the music and verify that they exhibit the typical characteristics of an auditory TRF, and perform well at predicting the neural responses to new stimuli.

**FIGURE 7:**
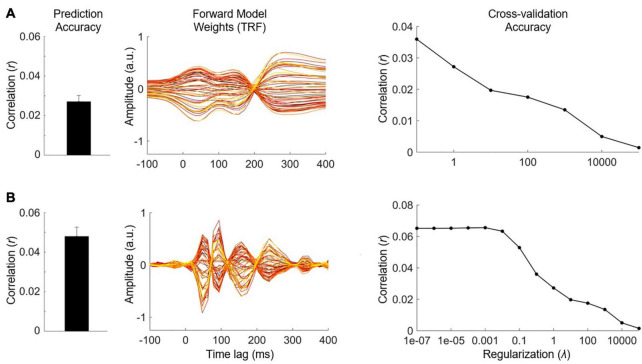
Interpreting TRF weights with sub-optimal regularization. **(A)** Forward model performance using a limited hyperparameter search in the range [10^–1^, 10^5^]. Left, mean prediction accuracy (Pearson’s *r*) using optimized regularization parameter, averaged across all EEG channels. Error bars indicate SEM across participants. Middle, optimized TRF weights for individual EEG channels. Darker colors indicate more posterior channels. Right, mean cross-validation accuracy as a function or regularization, averaged across participants. The maximal prediction score corresponded to a lambda value of 10^–1^. **(B)** The same data as in panel **(A)** for a more exhaustive hyperparameter search in the range [10^–7^, 10^5^]. This exhaustive search yielded a higher prediction accuracy than in **(A)**, corresponding to an optimal regularization value of 10^–3^.

The researcher decides to use the following parameters for preprocessing and modeling: (a) the EEG data is filtered between 1–30 Hz; (b) the TRFs are calculated using time lags between [–100, 400], which should be sufficient to capture the expected responses between about 20–250 ms; and (c) cross-validation is preformed across regularization values in the range [10^–1^, 10^5^]. The researcher first examines the average EEG prediction score based on the optimized models (Figure 7A, left). The values were not particularly high (∼0.025), but were significantly greater than zero. Thus, they decide to move forward with the next step and interpret the TRF weights (Figure 7A, middle). The resulting TRF exhibits two slow “components”: one starting before 0 ms (i.e., prior to stimulation) and terminating around 200 ms, the other is a sustained response with inverse polarity starting at 200 ms. While this may appear an interesting result, potentially reflecting the predictive nature of music perception, the dynamics of the response components appear to be much slower than previous reports. Thus, the researcher decides to double-check why that may be the case. In doing so, they realize that the optimal regularization value determined by the cross-validation procedure was always converging on the minimum value in the search interval (i.e., 10^–1^; Figure 7A, right). To resolve this, they then extended the parameter search to a broader interval of regularization values [10^–7^, 10^5^], this time verifying that the optimal regularization value was within that interval, and not at one of the extremes (Figure 7B, right). As a result, the average EEG prediction score was much higher (∼0.06; Figure 7B, left) and the TRFs appeared to have much faster dynamics, similar to previous work (Figure 7B, middle).

Here, the first pass at optimizing the model (Figure 7A) is a classic example of poor parameter selection and, as a result, a poorly fit model. The issue is not necessarily due to the choice of regularization values, but rather due to missing an initial check of the tuning curve. It is critical to ensure that the tuning curve has reached a global maximum before halting the parameter search.

## Conclusion

Linear modeling enables researchers to both understand and quantify neural processing of complex, continuous stimuli. Here, we provided a hypothetical experiment in which linear models were used to study phonological processing using natural speech in a clinical group with phonological deficits. This experiment highlights how comparing the neural prediction scores between two or more models can be used to quantify the unique contribution of specific stimulus features, such as phonological content, and isolate deficits in the processing of such features with great specificity. The mTRF-Toolbox provides the necessary tools to train and test multivariate stimulus-response models and address questions relating to stimulus feature encoding. Further details relating to the toolbox can be found in Crosse et al. (2016a).

Computational cognitive neuroscience is a rapidly advancing field, and it is utilizing and benefiting from ecological experimental design more and more. We have focused on linear modeling because it is straightforward and computationally efficient, producing models that are easily quantifiable and interpretable. Of course, there are other more complex and computationally intensive ways of analyzing neural responses to continuous stimuli. In particular, deep neural networks have shown promise for generating artificial neural responses that mimic the processing stages of sensory systems in the brain (Yamins and DiCarlo, 2016; Richards et al., 2019). Additionally, non-linear models generally outperform linear models in predicting neural data (but see Lalor et al., 2008; Crosse, 2011). However, they can be more difficult to interpret than linear models, and can be harder to compare across feature sets (Ivanova et al., 2021). Furthermore, it is not yet clear how much benefit non-linear models provide for modeling non-invasive population recordings (for discussion see Crosse et al., 2016a). Linear models provide a more direct bridge between the controlled experiment design of previous work and machine-learning-based analyses that work for experiments with continuous stimuli and multiple time-varying features of interest (Holdgraf et al., 2017). Because of this, linear models are an important part of the armamentarium for addressing questions in the field of applied cognitive neuroscience.

Throughout the paper, we discussed many of the issues relevant to modeling stimulus-response data for clinical studies. The following, are some important take-home points to bear in mind when analyzing clinical data:

• The amount of available data influences the quality of model fit, particularly at lower SNRs, and may determine the approach taken for training the model,
• Variability of neural responses in individuals influences model fit for subject-dependent analyses, potentially leading to group differences between cohorts with disparate levels of individual response variability,
• Variability of neural responses across individuals influences model fit for subject-independent analyses, potentially leading to group differences driven by disparate levels of heterogeneity,

With this in mind, the researcher should interpret the results of their modeling analysis with great care, taking into consideration the many factors that could influence model fit, and potentially drive any observed group differences. While it can sometimes be difficult to disentangle such factors from other neural processes of interest, we must endeavor to do so in order to maintain the integrity of such work which has the potential to reveal so much about how the human brain functions (and fails to function) in everyday life.

## Data Availability Statement

The latest version of the mTRF-Toolbox, including example code and data, can be found on GitHub (https://github.com/mickcrosse/mTRF-Toolbox). Code for the simulations used to create Figure 4 can also be found on GitHub (https://github.com/natezuk/mTRFToolbox_simulations). Resources from the 2021 CNSP-Workshop – which includes links to open-source tools, tutorials, public datasets and useful papers – can be found on the CNSP-Workshop website (https://cnspworkshop.net).

## Author Contributions

MC, NZ, GD, AN, SM, and EL wrote the draft of the manuscript. AN created all schematic figures. MC, NZ, GD, and AN contributed to data simulations, example analyses, and their figures. MC and AN contributed to the code and their implementation in the information boxes. All authors contributed to the manuscript revision and approved the final version.

## Conflict of Interest

MC was employed by the company Alphabet Inc. The remaining authors declare that the research was conducted in the absence of any commercial or financial relationships that could be construed as a potential conflict of interest.

## Publisher’s Note

All claims expressed in this article are solely those of the authors and do not necessarily represent those of their affiliated organizations, or those of the publisher, the editors and the reviewers. Any product that may be evaluated in this article, or claim that may be made by its manufacturer, is not guaranteed or endorsed by the publisher.

## Footnotes

1
https://github.com/mickcrosse/mTRF-Toolbox

2 Mahajan, Y., Crosse, M. J., Ching, A., Borg, S., Kim, J., and Davis, C. (in prep). *Test-retest Reliability of Encoding and Decoding Models of Natural Speech and its Sensitivity to Selective Attention in EEG.*

3 During EEG recording, the electric fields elicited by cortical neurons must first pass through several anatomical layers (e.g., cerebrospinal fluid, the meningeal layers, skull bone, periosteum, and skin tissues), before reaching the electrode surface. These materials act as spatial filters and attenuate the EEG signal, particularly above 25 Hz.

4 In neural systems, the natural flow of information propagates forward from stimulus to brain, or downstream from one brain region to another. Because such models describe how information is encoded in the neural activity at a given sensor or brain region, they are also known as encoding models.

5 This is achieved by summing the covariance matrices across all trials only once at the beginning, storing the trial-specific covariance matrices in memory and iteratively subtracting each of them from the sum on the corresponding fold of the cross-validation procedure. To be more memory efficient, the trial-specific covariance matrices can instead be recomputed on every fold via the ‘fast’ parameter. However, the extra computations will make the cross-validation procedure slightly longer to run.

6
https://github.com/mickcrosse/PERMUTOOLS

7 The amount of regularization should in theory be the same for all subjects, assuming the datasets are recorded, amplified and subsequently normalized in the same manner. Subjects that require more regularization typically have a lower SNR, thus shrinking the model weights to limit their contribution toward prediction is beneficial. While this may improve their overall prediction score, it does not necessarily make the TRF estimate more “brain-like” when there is an insufficient amount of data or data with low SNR.
